# Associations between breastfeeding, childhood BMI and pubertal onset: findings from a prospective cohort study

**DOI:** 10.1016/j.ajcnut.2026.101208

**Published:** 2026-01-27

**Authors:** Maria J Ramirez-Luzuriaga, Madhumita Sinha, Robert L Hanson

**Affiliations:** Diabetes Epidemiology and Clinical Research Section, National Institute of Diabetes and Digestive and Kidney Diseases, National Institutes of Health, Phoenix, AZ, United States

**Keywords:** exclusive breastfeeding, breastfeeding duration, pubertal timing, adolescent growth spurt, child BMI

## Abstract

**Background:**

Early onset of puberty, often characterized by an accelerated linear growth spurt, is a recognized risk factor for a range of metabolic and cardiovascular conditions. Although increased breastfeeding has been associated with later onset of puberty, the potential mediating role of prepubertal BMI in these associations remains poorly understood.

**Objectives:**

This study aimed to examine the longitudinal associations of breastfeeding duration with adolescent growth parameters, including pubertal timing, and to assess whether prepubertal BMI mediates these associations.

**Methods:**

Adolescent growth parameters were estimated from the height growth trajectories of 613 participants (312 females and 301 males) across 6 cohorts in the Environmental Influences on Child Health Outcomes Program. These parameters were derived by fitting the Preece–Baines growth model, a parametric growth curve fitted to longitudinal height data, in participants with ≥3 height measurements spanning the whole period of growth. Linear regression models were used to examine associations of breastfeeding duration with adolescent growth parameters. Mediation analysis was conducted to explore whether prepubertal BMI mediated the association between breastfeeding and pubertal timing.

**Results:**

After adjusting for socioeconomic, maternal, and infant characteristics, children exclusively breastfed for ≥3 mo reached age at peak velocity and age at maturation later than those who were not exclusively breastfed [*β* = 0.32 y; 95% confidence interval (CI): 0.05, 0.60, and *β* = 0.30 y; 95% CI: 0.04, 0.56, respectively]. In adjusted models, each additional 3 mo of any breastfeeding was associated with a later age at take-off (*β* = 0.07 y; 95% CI: 0.00, 0.15), and later age at peak velocity (*β* = 0.11 y; 95% CI: 0.01, 0.20). Prepubertal BMI did not significantly mediate these associations.

**Conclusions:**

Exclusive breastfeeding and longer breastfeeding duration were associated with later onset of puberty in boys and girls. Prepubertal BMI did not mediate the observed associations.

## Introduction

Early onset of puberty that is often characterized by accelerated linear growth and early skeletal maturation, is a well-established risk factor for a range of metabolic and cardiovascular conditions [1]. Extensive research has linked the early onset of puberty to higher BMI [[1], [2], [3]], as well as increased risks of hypertension [4,5], type 2 diabetes [[5], [6], [7]], insulin resistance [2,3,8], and greater incidence of cardiovascular disease [4], breast, ovarian, and testicular cancers in adulthood [[9], [10], [11]].

Growing evidence indicates that early-life factors such as birth weight [[12], [13], [14]], and infant growth patterns [12,15] influence pubertal timing. These findings suggest that certain in utero biological and environmental influences may impact fetal growth, which, in turn, can affect subsequent developmental trajectories. Early-life nutrition also plays a central role in human growth and development. Although previous studies have examined the effect of breastfeeding practices on pubertal onset, their findings have been inconsistent [[16], [17], [18], [19], [20]]. Most of these studies have focused on female cohorts [17,18,20] and have been limited by the lack of readily available pubertal indicators, as some have relied on subjective measures to define the onset of puberty [16,18]. One of the hallmarks of puberty is the growth spurt in height, a phase of rapid physical growth driven primarily by hormonal changes [21]. Girls typically experience their growth spurt earlier than boys, with growth generally slowing down after puberty concludes. Tracking height changes over time enables the estimation of the adolescent growth spurt, offering an objective measure of pubertal timing in both males and females. One study in Japan examined the relationship between breastfeeding practices and pubertal timing in both males and females by analyzing the adolescent growth spurt [19]. This study used semiparametric growth measures, focusing on age at peak velocity, but did not investigate other growth parameters, which have also been strongly associated with chronic disease risk [7].

In addition, breastfeeding, particularly when feeding directly at the breast, has generally been linked to less weight gain during childhood [[22], [23], [24]]. Overweight and obesity in childhood are known risk factors for early pubertal onset [14,25]. However, the potential mediating role of childhood weight on the association between breastfeeding and pubertal timing remains poorly understood.

The present study aimed to: *1*) examine the longitudinal associations between breastfeeding duration and adolescent growth parameters, including pubertal timing, in boys and girls; and *2*) assess whether prepubertal BMI mediates the association between breastfeeding duration and pubertal outcomes. To analyze pubertal development in both males and females, we apply the Preece–Baines growth model, a parametric model for assessing growth in height that captures a wide range of developmental parameters [26]. This model is well-validated, and it has been widely used to study the adolescent growth spurt [27,28].

## Methods

### Study participants

The present study used data from the second release of the Environmental Influences on Child Health Outcomes (ECHO)-Wide Cohort Study [29], part of the ECHO Program. The ECHO Program was funded in 2016 by the NIH to investigate how environmental exposures from preconception through early childhood influence child health and developmental outcomes [30,31]. The ECHO-Wide Cohort study comprises 69 pediatric cohorts across the United States [29]. Enrollment started in the 1980s at different life stages, most often either during pregnancy or at birth and is ongoing. In 2019, the ECHO-Wide cohorts adopted the standardized ECHO-Wide data collection protocol. Extant data obtained before the adoption of the standardized ECHO-Wide protocol were harmonized across cohorts [29].

Optimal estimation of growth parameters requires height measurements taken before, during, and after the adolescent growth spurt. The inclusion criteria for this study consisted of participants with ≥3 height measurements spanning the entire childhood and adolescence growth period (≥1 measure at ages 1–9 y, ≥1 measure at ages 10–15 and ≥1 measure at ages ≥16 y), along with available breastfeeding data. The final sample for analyses included 613 participants (312 females and 301 males) from 6 cohorts who met these criteria (Supplementary Figure 1)**.** Over two-thirds of participants contributed ≥5 repeated measures across the growth period, enabling robust curve estimation (Supplementary Table 1). Three cohorts accounted for 99% of the sample. Demographic characteristics of these cohorts are presented in Supplementary Table 2, with included participants showing similar characteristics to excluded individuals within the same cohorts (Supplementary Table 3).

The ECHO-Wide Cohort Data Collection Protocol was approved by the central Institutional Review Board (IRB) or by the IRBs of record for individual cohorts. Written informed consent was obtained from all participants in accordance with local institutional review boards and ECHO Program protocols. The present analyses were based on deidentified data obtained from the National Institute of Child Health and Development’s Data and Specimen Hub with approval from the Data Access Committee.

### Measures

#### Exposure: breastfeeding

Given that this study includes data collected before the adoption of the standardized ECHO-Wide Cohort Protocol, breastfeeding data were assessed differently across cohorts. These data were subsequently harmonized into the summary variables used in this analysis. The available summary breastfeeding variables that were used for this study were reported exclusive breastfeeding through age 3 mo and reported duration of any breastfeeding. Exclusive breastfeeding through age 3 mo was defined as the intake of breast milk excluding formula and complementary foods and was treated as a binary variable (yes/no). This variable definition confirmed the absence of formula but not the absence of complementary foods and supplements, as it allowed for missing data on foods and supplements (i.e., if missing these were assumed to be absent). Additionally, it did not capture information on pumped milk. Any breastfeeding was defined as the intake of breast milk, regardless of the consumption of other foods or drinks. Duration of any breastfeeding was defined as the infant’s age in weeks when any breastfeeding had been permanently stopped and was examined as a continuous variable. To help interpretability, continuous breastfeeding was scaled to 12 wk to express the results per each 3-mo difference in breastfeeding duration. For descriptive purposes and for graphical presentation of growth curves, we additionally categorized breastfeeding duration into 3 groups: <12 wk, 12–24 wk and >24 wk.

#### Outcome

The primary outcomes of this study are growth parameters of the adolescent growth spurt, including pubertal timing, which are derived from longitudinal height measurements obtained through both research visits and medical records. Biological milestones of the growth process include the timing and velocity of the adolescent growth spurt [32]. Participant-specific height trajectories were modeled using the Preece–Baines growth model I [26,33]. Details of the estimation procedure are shown in Supplementary Methods. Briefly, the growth model uses a parametric nonlinear mixed model to estimate 5 parameters of growth in height from childhood to adulthood, which are then used to calculate important biological features of the growth process for each participant. These features are: *1*) age at the onset of the adolescent growth spurt, i.e., age at take-off, *2*) velocity at take-off, *3*) height at take-off, *4*) age at the time of the maximum growth spurt, i.e., age at peak height velocity, *5*) peak height velocity, *6*) height at peak velocity, *7*) age at maturation, taken at the age at which the predicted growth velocity fell below 1 cm/y [34], and *8*) attained adult height. To assess the robustness of our modeling approach to sparse data with as few as 3 examinations, we conducted a cross-validation analyses in which data from individuals with ≥5 examinations were selectively masked so that only 3 examinations were used (see Supplementary Methods for details). The results of this analysis found a high degree of consistency when using 3 examinations compared with results from ≥5 examinations (Supplementary Table 4).

#### Confounders/mediators

We identified potential confounders as variables associated with both breastfeeding practices and adolescent growth parameters but not in the putative causal pathways between them. These included child characteristics (sex, race, birthweight, and birth length) and maternal characteristics (education, income, age at delivery, height, prepregnancy BMI, and weight gain during pregnancy). These variables have been used in previous publications that analyzed the association between breastfeeding and pubertal onset [17]. We hypothesized that child BMI at ages 5 and 10 y may act as mediators on the causal pathway from breastfeeding practices to adolescent growth. The following pediatric and birth characteristics were obtained from caregiver reports or medical records: child’s date of birth, cohort id, sex, birthweight (in grams), birth length (in cm), parent-reported race (American Indian or Alaska Native, Asian, Black, Native Hawaiian or Pacific Islander, White, and multiple races), and parent-reported ethnicity (non-Hispanic or Latino and Hispanic or Latino). Because of the small sample size, we combined the number of children who identified as American Indian or Alaska Native, Asian, Native Hawaiian or Pacific Islander, and multiple races into a single category. We additionally obtained the child’s BMI *z*-scores when age at examination was closest to age 5 and age 10, available from derived/summary data. In addition, the following maternal characteristics were obtained from maternal or caregiver reports or medical records: mother’s highest educational level completed (less than high school, high school degree, <4-y college degree or higher); annual household income during pregnancy (<$30,000 or ≥$30,000); maternal age at delivery (in years); maternal height (in cm) prepregnancy BMI (assessed from 12 mo before conception through the end of the first trimester); adherence to weight change guidelines during pregnancy based on 2009 Institute of Medicine (IOM) recommendations (gained less than the recommendation, met the recommendation, gained more than the recommendation); and gestational age at delivery (in weeks).

#### Analytical sample

Our analytical sample consisted of participants with available data on exclusive breastfeeding or breastfeeding duration, along with a minimum of 3 height measurements spanning the entire growth period required to derive growth spurt parameters. A total of 528 participants had available data on exclusive breastfeeding and growth parameters, whereas 374 participants had data on any breastfeeding duration and growth parameters. The overlap between these 2 samples comprised 289 participants. To ensure consistency in the analyses, missing data on breastfeeding variables were addressed using multiple imputation (see below). The proportion of missing data was 38.9% for breastfeeding duration and 13.2% for exclusive breastfeeding. The final analytical sample included 613 participants. Results from complete-case analyses are also presented as sensitivity analyses.

### Statistical analyses

To evaluate the associations of exclusive breastfeeding through age 3 mo and any breastfeeding duration with each parameter of adolescent growth, we used linear regression models adjusting for relevant known confounders. Analyses were conducted both in pooled samples and stratified by sex. Model 1 accounted for basic characteristics, including the child’s date of birth, race, and cohort id, with cohort id included as a fixed-effect term to account for clustering within cohorts. In model 2, socioeconomic, maternal and birth characteristics were included as covariates (i.e., maternal education, annual household income during pregnancy, maternal age at delivery, maternal height, maternal pregestational BMI, total gestational weight change, gestational age at delivery, birthweight, and birth length). Model 3 additionally adjusted for the child’s BMI z-score at age 5. Pooled models were further adjusted for sex. Interaction between each breastfeeding variable and the child’s sex was tested by including product terms. Equality of parameter estimates across sexes was assessed using the Wald χ^2^ test for interaction, with a *P* value < 0.05 indicating statistical significance. To assess the influence of breastfeeding on childhood weight, we examined the associations of exclusive breastfeeding and any breastfeeding duration with childhood BMI *z*-scores at ages 5 and at 10 y using linear regression models, adjusting for potential confounders. To further test the robustness of our results we examined associations of exclusive breastfeeding and breastfeeding duration with growth parameters, stratified by number of exams used to derive the Preece–Baines growth curves. In all models, we used multiple imputation by chained equations to address the missing data. Specifically, we performed 50 imputations with 100 iterations. The variables with missing data included breastfeeding duration, exclusive breastfeeding (categorized into 2 groups), maternal age at delivery, maternal pregestational BMI, maternal height, maternal education (categorized into 3 groups), annual household income during pregnancy (categorized into 2 groups), gestational age at birth, birth length, child BMI z-scores at ages 5 and 10, and total gestational weight gain (categorized into 3 groups). The proportion of missing data addressed through imputation is shown in Supplementary Table 5. The imputation model assumed that, conditional on covariates, data were missing at random (MAR). Analyses of missing data patterns supported the MAR model (Supplementary Methods and Supplementary Table 6). The imputation model incorporated all variables that predicted missingness, and all variables included in the analysis’s models.

We present nominal *P* values and confidence intervals (CIs) for each of the 8 outcomes without correction for multiple comparisons. Although this approach avoids the increase in type II error associated with correction for multiple comparisons, it can increase type I error. To assess overall type I error, accounting for correlations among adolescent growth parameters, we also applied the Brown–Kost test, a method which combines nonindependent *P* values into a single aggregated test of the global null hypothesis of no association between breastfeeding and any of the 8 outcomes [35,36]. This approach allowed us to evaluate the overall evidence across correlated outcomes while preserving the interpretability of each individual outcome.

We used mediation analysis methods to examine whether BMI in childhood mediates the association of breastfeeding duration and exclusive breastfeeding with pubertal development outcomes [37]. In these models, the BMI *z*-score closest to age 5 was used as the potential mediator, as it typically reflects adiposity before the onset of the growth spurt. For completeness, we also present mediation analyses using the BMI *z*-score closest to age 10. These analyses were conducted with the CAUSALMED procedure in SAS (SAS Institute, Inc.), which uses a counterfactual-based analysis to assess causal parameters [38]. The overall effect size (*β* coefficient) is partitioned into natural direct (unmediated) and indirect (mediated) effects, and percentage mediation is estimated from the ratio of the indirect to the total effect size. Models adjusted for child’s date of birth, race (American Indian or Alaska Native, Asian, Black, Native Hawaiian or Pacific Islander, White, and multiple races), mother’s highest educational level completed (less than high school, high school degree, <4-y college degree or higher), annual household income during pregnancy (<$30,000 or ≥$30,000), maternal age at delivery (in years), maternal height (in cm), maternal pregestational BMI (assessed from 12 mo before conception through the end of the first trimester), adherence to weight change guidelines during pregnancy based on 2009 IOM recommendations (gained less than the recommendation, met the recommendation, gained more than the recommendation), gestational age at delivery (in weeks), birthweight (in grams), and birth length (in cm). CIs were calculated by a bootstrap method using 2000 resamples and 20 iterations. All analyses were performed using the SAS (version 9.4; SAS Institute). A 2-sided *P <* 0.05 was considered statistically significant.

## Results

Of 613 participants included in the analysis, 312 (50.9%) were girls and 301 (49.1%) boys. Most legal guardian or parents of participants self-reported race as either White (50.4%) or Black (44.5%). Only 5.1% self-reported as American Indian or Alaska Native, native Hawaiian or Pacific Islander, multiple races, or another race. The proportion of exclusive breastfeeding through age 3 mo was 13.5%. Approximately half of the participants breastfed for <12 wk, whereas one-quarter breastfed for >24 wk (Table 1). As expected, mean age at take-off of the adolescent growth spurt started 1.3 y earlier in females than in males (at 7.7 y in females, compared with 9.0 y in males). Mean age at peak velocity was reached ∼3 y after the onset of the growth spurt; at ages 10.5 y in females and 12.8 y in males. At this age, boys and girls grew ∼ 7 cm/y. After having reached a peak, the growth velocity decreases rapidly signaling the end of the growth cycle. Age at maturation, near final height, occurred at around age 14.9 y in girls and 17.0 y in boys (Supplementary Table 7).

**TABLE 1:** Selected study characteristics (mean ± SD or %) by breastfeeding variables^1^

	Exclusive breastfeeding through age 3 mo		Duration of any breastfeeding		
	Yes (*n* = 83)	No (*n =* 530)	<12 wk (*n =* 302)	12–24 wk (*n =* 150)	>24 wk (*n =* 161)
Maternal characteristics					
Pregestational BMI^2^ (kg/m^2^)	25.3 ± 6.48	27.4 ± 7.44	27.7 ± 7.75	26.0 ± 6.36	27.1 ± 7.37
Maternal age at delivery (y)	32.9 ± 6.03	32.2 ± 6.90	32.1 ± 6.95	32.5 ± 6.17	32.3 ± 7.02
Maternal height (cm)	163.6 ± 7.96	163.8 ± 7.27	163.7 ± 7.34	163.8 ± 7.27	164.0 ± 7.52
Weight change during pregnancy (based on 2009 IOM recommendation) (%)					
Gained less than the recommendation	86.8	78.5	78.8	84.7	76.4
Met the recommendation	4.82	6.60	5.96	7.33	6.21
Gained more than recommendation	8.43	14.9	15.2	8.00	17.4
Gestational age at delivery (wk)	38.5 ± 2.04	38.2 ± 2.28	37.9 ± 2.39	38.7 ± 1.95	38.3 ± 2.18
Mother’s highest educational level completed (%)					
Less than high school	45.8	64.9	69.2	46.7	64.0
High school degree	2.41	8.68	8.61	5.33	8.70
Some college	51.8	26.4	22.2	48.0	27.3
Annual household income during pregnancy (%)					
<$30.000	51.8	69.2	73.5	48.0	72.1
≥$30.000	48.2	30.8	26.5	52.0	27.9
Offspring characteristics					
Male (%)	61.4	47.2	48.0	49.3	50.9
Birthweight (g)	3555 ± 543	3342 ± 577	3319 ± 597	3442 ± 556	3403 ± 551
Birth length (cm)	50.7 ± 2.90	50.2 ± 3.30	50.2 ± 3.46	50.1 ± 2.92	50.7 ± 3.12
Age at examination closest to age 5	6.44 ± 1.47	5.66 ± 1.29	5.63 ± 1.28	6.47 ± 1.40	5.43 ± 1.21
BMI *z*-score at age 5 y	0.39 ± 1.09	0.61 ± 1.13	0.65 ± 1.12	0.54 ± 1.09	0.50 ± 1.16
Age at examination closest to age 10	12.7 ± 0.56	12.6 ± 0.92	12.6 ± 0.94	12.7 ± 0.53	12.6 ± 1.00
BMI *z*-score at age 10 y	0.39 ± 1.08	0.78 ± 1.14	0.85 ± 1.11	0.54 ± 1.15	0.66 ± 1.16
Race (%)					
White	75.9	46.4	44.7	60.0	52.2
Black	15.7	49.1	50.1	32.0	44.1
Other	8.43	4.53	4.30	8.00	3.73
Ethnicity (%)					
Not Hispanic or Latino	94.0	96.0	96.4	93.3	96.9
Hispanic or Latino	6.00	3.96	3.64	6.67	3.11

Abbreviation: IOM, Institute of Medicine.

1 Values are mean ± SD or % unless specified otherwise.

2 The maternal prepregnancy BMI that was assessed from 12 mo before conception through the end of the first trimester (<13 wk gestational age) was used.

**FIGURE 1:**
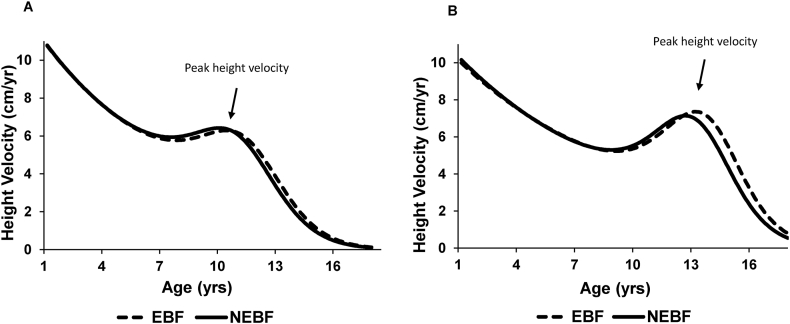
Predicted mean height growth velocity by EBF and NEBF categories. (Adjusted for child’s date of birth, race, and cohort id.) (A) Females. (B) Males. In females, the mean age at peak velocity was 10.9 y in EBF and 10.4 y in NEBF; in males, the corresponding values were 13.4 y and 12.8 y. Growth parameters were derived by fitting the Preece–Baines growth model I, a parametric growth curve fitted to longitudinal data. EBF, exclusive breastfeeding; NEBF, nonexclusive breastfeeding.

Figure 1 shows predicted mean height growth velocities by exclusive breastfeeding categories in females and males. In both sexes, growth curves for individuals exclusively breastfed through 3 mo are shifted to the right, indicating a later onset of puberty, as reflected by later age at take-off, age at peak velocity and age at maturation, compared with those who were not exclusively breastfed. Table 2 shows associations of exclusive breastfeeding through 3 mo of age with adolescent growth parameters. In models adjusted for sex, socioeconomic, maternal, and infant characteristics, exclusive breastfeeding through 3 mo of age (compared with no exclusive breastfeeding) was associated with later age at peak velocity (*β* = 0.32 y; 95% CI: 0.05, 0.60) and later age at maturation (*β* = 0.30 y; 95% CI: 0.04, 0.56). These associations were slightly attenuated but remained statistically significant after further adjustment for BMI *z*-score at age 5 y (Table 2). Similar results were observed in the complete-case analysis (Supplementary Table 8).

**TABLE 2:** Associations of exclusive breastfeeding through 3 mo of age (vs. no exclusive breastfeeding) with adolescent growth parameters^1^

	Pooled (*n =* 613)			Females (*n =* 312)			Males (*n =* 301)		
	Model 1	Model 2	Model 3	Model 1	Model 2	Model 3	Model 1	Model 2	Model 3
Age at take-off (y)	0.23 (0.03, 0.42)	0.21 (–0.00, 0.42)	0.19 (–0.01, 0.40)	0.25 (–0.09, 0.60)	0.26 (–0.09, 0.62)	0.25 (–0.10, 0.60)	0.23 (0.00, 0.46)	0.19 (–0.06, 0.45)	0.17 (−0.08, 0.42)
Age at peak velocity (y)	0.35 (0.09, 0.61)	0.32 (0.05, 0.60)	0.28 (0.02, 0.54)	0.30 (−0.13, 0.75)	0.30 (−0.15, 0.75)	0.27 (−0.15, 0.69)	0.43 (0.12, 0.74)	0.39 (0.04, 0.74)	0.34 (0.00, 0.67)
Age at maturation (y)	0.34 (0.10, 0.59)	0.30 (0.04, 0.56)	0.27 (0.02, 0.52)	0.29 (–0.06, 0.66)	0.30 (–0.06, 0.68)	0.28 (–0.06, 0.64)	0.41 (0.08, 0.75)	0.33 (–0.03, 0.71)	0.29 (–0.06, 0.66)
Velocity at take-off (cm/y)	–0.08 (–0.25, 0.08)^2^	–0.08 (–0.26, 0.08)^2^	–0.07 (–0.24, 0.10)^2^	–0.14 (–0.46, 0.17)	–0.12 (–0.44, 0.19)	–0.10 (–0.41, 0.20)	–0.05 (–0.23,0.11)	–0.07 (–0.27, 0.11)	–0.05 (–0.24, 0.12)
Peak velocity, (cm/y)	0.07 (–0.15, 0.30)^2^	0.08 (–0.16, 0.33)^2^	0.07 (–0.18, 0.32)^2^	–0.16 (–0.48, 0.14)	–0.16 (–0.48, 0.15)	–0.16 (–0.48, 0.15)	0.23 (–0.10, 0.57)	0.26 (–0.10, 0.64)	0.22 (–0.15, 0.60)
Attained height (cm)	1.66 (–0.18, 3.52)	1.15 (–0.62, 2.94)	1.17 (–0.61, 2.97)	0.66 (–2.08, 3.41)	0.91, (–1.64, 3.48)	0.94 (–1.62, 3.51)	2.52 (0.00, 5.03)	1.58 (–0.94, 4.11)	1.60 (–0.93, 4.14)
Height at take-off (cm)	0.91 (–0.82, 2.65)	0.46 (–1.26, 2.19)	0.60 (–1.14, 2.35)	1.21 (–1.61, 4.03)	1.43 (–1.31, 4.18)	1.51 (–1.26, 4.29)	0.77 (–1.42, 2.97)	–0.05 (–2.32, 2.20)	0.18 (–2.04, 2.42)
Height at peak velocity (cm)	1.62 (–0.02, 3.26)	1.12 (–0.46, 2.71)	1.11 (–0.47, 2.71)	1.09 (–1.28, 3.47)	1.24 (–1.01, 3.49)	1.22 (–1.03, 3.48)	2.20 (–0.04, 4.46)	1.38 (–0.87, 3.63)	1.42 (–0.83, 3.68)

Abbreviation: CI, confidence interval.

1 Estimates are β coefficients (95% CI) from linear regression models. Model 1 accounts for child’s date of birth, race, and cohort id. Model 2 additionally accounts for maternal education, annual household income during pregnancy, maternal age at delivery, maternal height, maternal prepregnancy BMI, total gestational weight change, gestational age at delivery, birthweight, and birth length. Model 3 additionally accounts for child BMI *z*-score at age 5. Pooled models additionally adjust for sex. Multiple imputation techniques were used for missing covariates.

2 *P* < 0.05 for interaction of breastfeeding duration with sex.

Table 3 presents the associations between the duration of any breastfeeding (per 3-mo increment) and adolescent growth parameters. In pooled models adjusting for socioeconomic, maternal, and infant characteristics, each additional 3-mo of breastfeeding was associated with greater age at take-off (*β* = 0.07 y; 95% CI: 0.00, 0.15), and later age at peak velocity (*β* = 0.11 y; 95% CI: 0.01, 0.20). These associations were slightly attenuated after additional adjustment for BMI *z*-scores at age 5 y but remained statistically significant for age at peak velocity (*β* = 0.09 y; 95% CI: 0.00, 0.18). Associations appeared stronger in females than in males; however, no statistically significant sex interaction was observed in the pooled models (Table 3). Results from complete-case analysis were slightly stronger than those obtained using imputed data (Supplementary Table 9).

**TABLE 3:** Associations of any breastfeeding duration (per 3-mo increase) with adolescent growth parameters^1^

	Pooled (*n =* 613)			Females (*n =* 312)			Males (*n* = 301)		
	Model 1	Model 2	Model 3	Model 1	Model 2	Model 3	Model 1	Model 2	Model 3
Age at take-off (y)	0.08 (0.02, 0.15)	0.07 (0.00, 0.15)	0.07 (–0.00, 0.14)	0.13 (0.04, 0.23)	0.12 (0.01, 0.23)	0.11 (0.00, 0.22)	0.04 (–0.03, 0.17)	0.03 (–0.05, 0.12)	0.02 (–0.06, 0.11)
Age at peak velocity (y)	0.12 (0.04, 0.19)	0.11 (0.01, 0.20)	0.09 (0.00, 0.18)	0.17 (0.05, 0.29)	0.16 (0.02, 0.29)	0.14 (0.01, 0.27)	0.06 (–0.03, 0.16)	0.06 (–0.06, 0.18)	0.03 (–0.07, 0.15)
Age at maturation (y)	0.09 (0.02, 0.17)	0.08 (–0.00, 0.17)	0.06 (–0.01, 0.15)	0.14 (0.04, 0.23)	0.13 (0.02, 0.24)	0.12 (0.01, 0.23)	0.05 (–0.05, 0.16)	0.03 (–0.09, 0.17)	0.01 (–0.11, 0.14)
Velocity at take-off (cm/y)	–0.04 (–0.10, 0.00)^2^	–0.04 (–0.10, 0.01)^2^	–0.03 (–0.09, 0.01)^2^	–0.10 (–0.18, –0.01)	–0.09 (–0.19, 0.00)	–0.08 (–0.17, 0.01)	0.00 (–0.05, 0.05)	–0.00 (–0.06, 0.05)	0.00 (–0.05, 0.06)
Peak velocity (cm/y)	–0.00 (–0.08, 0.07)^2^	0.00 (–0.07, 0.09)^2^	0.00 (–0.08, 0.08)^2^	–0.08 (–0.17, –0.00)	–0.07 (–0.17, 0.02)	–0.07 (–0.17, 0.02)	0.06 (–0.04, 0.18)	0.09 (–0.03, 0.21)	0.07 (–0.05, 0.20)
Attained height (cm)	0.44 (–0.12, 1.01)	0.30 (–0.27, 0.88)	0.31 (–0.26, 0.89)	0.18 (–0.59, 0.97)	0.16 (–0.61, 0.95)	0.18 (–0.59, 0.96)	0.61 (–0.20, 1.42)	0.38 (–0.45, 1.23)	0.40 (–0.45, 1.25)
Height at take-off (cm)	0.44 (–0.09, 0.98)	0.26 (–0.28, 0.82)	0.35 (–0.19, 0.90)	0.53 (–0.24, 1.31)	0.41 (–0.37, 1.21)	0.48 (–0.30, 1.26)	0.29 (–0.41, 1.01)	0.03 (–0.70, 0.77)	0.15 (–0.57, 0.88)
Height at peak velocity (cm)	0.56 (0.06, 1.06)	0.41 (–0.09, 0.92)	0.41 (–0.09, 0.93)	0.50 (–0.15, 1.17)	0.43 (–0.22, 1.08)	0.42 (–0.23, 1.08)	0.55 (–0.18, 1.28)	0.33 (–0.43, 1.09)	0.35 (–0.41, 1.12)

Abbreviation: CI, confidence interval.

1 Estimates are *β* coefficients (95% CI) from linear regression models. Model 1 accounts for child’s date of birth, race, and cohort id. Model 2 additionally accounts for maternal education, annual household income during pregnancy, maternal age at delivery, maternal height, maternal prepregnancy BMI, total gestational weight change, gestational age at delivery, birthweight, and birth length. Model 3: additionally accounts for child BMI *z*-score at age 5. Pooled models additionally adjust for sex. Multiple imputation techniques were used for missing covariates.

2 *P* < 0.05 for interaction of breastfeeding duration with sex.

**FIGURE 2:**
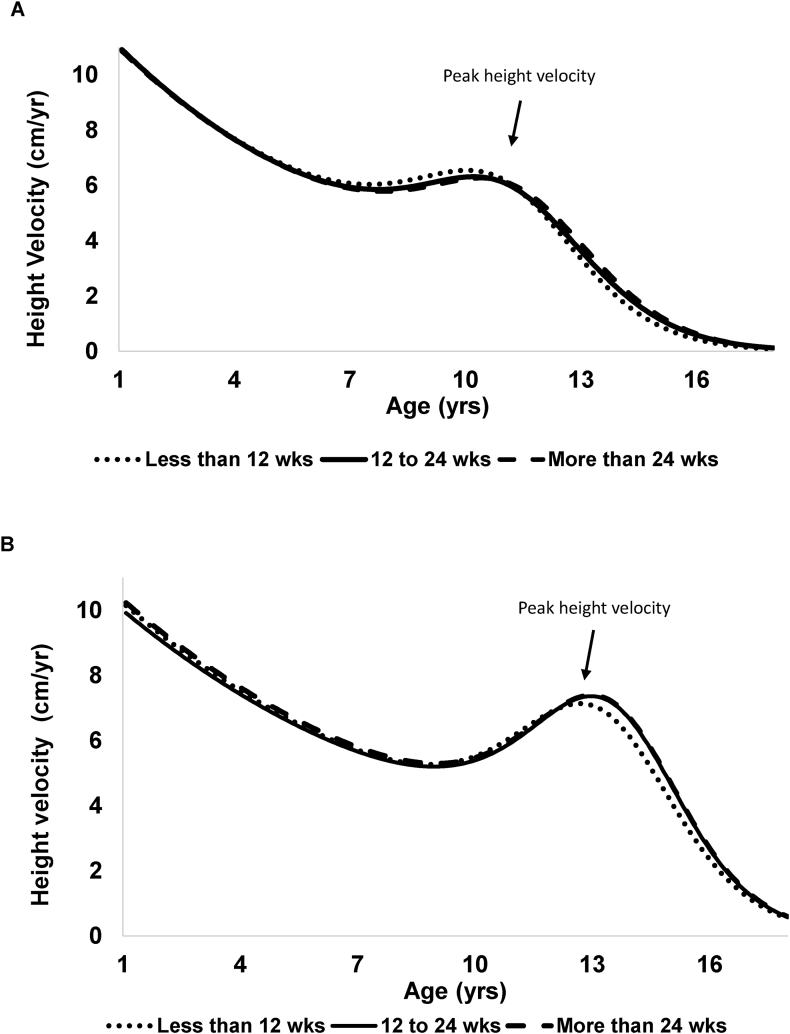
Predicted mean height growth velocity by breastfeeding duration categories (<12 wk, 12–24 wk, >24 wk).^1^ (A) Females. (B) Males. In females, mean age at peak velocity was 10.2 y for <12 wk, 10.7 y for 12–24 wk and 10.9 y for >24 wk; in males, the corresponding values were 12.8 y, 12.9 y and 13.1 y. Growth parameters were derived by fitting the Preece–Baines growth model I, a parametric growth curve fitted to longitudinal height data. ^1^Adjusted for child’s date of birth, race, and cohort id.

Figure 2 shows predicted mean height growth velocities by breastfeeding duration categories in females and males. In both sexes, growth curves for individuals breastfed for <12 wk were shifted to the left, indicating an earlier onset of puberty, reflected by earlier age at take-off, age at peak velocity and age at maturation, compared with those breastfed for 12 to 24 wk and >24 wk.

Although not statistically significant, exclusive breastfeeding was associated with lower child BMI *z*-scores at age 5 y (Supplementary Table 10). Stronger and significant associations were found later in childhood, at age 10 y. Specifically, in models adjusted for sex and age at measurement, exclusive breastfeeding (compared with no exclusive breastfeeding) was associated with a 0.40-unit lower BMI z-score (95% CI: –0.69, –0.10). This association was attenuated after further adjustment for maternal characteristics (*β* = –0.26, 95% CI: –0.57, 0.03). Longer duration of any breastfeeding was also associated with lower BMI z-scores at both ages 5 and 10 y, but these associations were smaller in magnitude (Supplementary Table 10).

The Brown–Kost global test yielded statistically significant *P* values for associations of exclusive breastfeeding (*P =* 0.0177) and breastfeeding duration (*P =* 0.0108) with growth parameters, supporting rejection of the global null hypothesis in favor of the alternative of association of breastfeeding with ≥1 growth outcome. When stratified by the number of examinations used to derive Preece–Baines growth curves, associations remained consistent for most parameters, particularly those reflecting the timing and tempo of the adolescent growth spurt (Supplementary Tables 11 and 12). Parameter estimates did not differ significantly between participants with fewer than 5 examinations and those with ≥5.

### Quantifying the influence of childhood BMI

We report mediation results for associations where the total effect between breastfeeding and adolescent growth spurt parameters was statistically significant. In causal mediation models, we found little evidence that BMI *z*-scores at age 5 or 10 y mediated the association between exclusive breastfeeding through 3 mo age and adolescent growth parameters (Table 4). The proportion of the effect mediated by BMI *z*-scores at age 5 y ranged from 6.7% to 14.9%, with none reaching statistical significance. Similarly, in adjusted mediation models examining breastfeeding duration, BMI *z*-scores at age 5 y accounted for only a small proportion of the association with growth parameters; with proportions ranging from 7.6% to 15.7%; again, none were statistically significant.

**TABLE 4:** Percent mediation through BMI for age-*z* scores in childhood for associations of breastfeeding with adolescent growth parameters

	Exclusive breastfeeding through age 3 mo				Breastfeeding duration			
	% Mediation through BMI at age 5 y	*P* value	% Mediation through BMI at age 10 y	*P* value	% Mediation through BMI at age 5 y	*P* value	% Mediation through BMI at age 10 y	*P* value
Age at take-off	6.7 (–8.49, 38.8)	0.224	11.5 (–20.6, 46.3)	0.244	7.6 (–12.9, 34.5)	0.214	11.9 (–28.8, 61.7)	0.124
Age at peak velocity	14.9 (–14.2, 40.8)	0.204	16.0 (–10.7, 38.9)	0.184	15.7 (–21.2, 44.5)	0.134	20.6 (–31.6, 66.9)	0.134
Age at maturation	10.7 (–7.18, 45.6)	0.184	13.5 (–13.1, 39.8)	0.184	14.3 (–10.6, 46.4)	0.124	21.2 (–4.22, 95.7)	0.074

Analyses based on counterfactuals (PROC CAUSALMED). Values are percentages (95% CI). All models accounted for child’s sex, date of birth, race, cohort id, maternal education, annual household income during pregnancy, maternal age at delivery, maternal height, maternal prepregnancy BMI, total gestational weight change, gestational age at delivery, birthweight, and birth length.

Abbreviation: CI, confidence interval.

## Discussion

In this study, we examined the association between breastfeeding and adolescent growth parameters in a diverse (50% White and 45% Black) sample from the United States ECHO-wide cohort study. Our findings suggest that breastfeeding, whether measured as exclusive breastfeeding or total duration, was associated with later pubertal onset, as reflected by positive associations with age at take-off, age at peak velocity, and age at maturation. In adjusted models, children exclusively breastfed through age 3 mo experienced ∼4 mo delay in age at peak velocity and 3 mo delay in age at maturation, compared with those not exclusively breastfed. These associations were stronger in males, although sex differences were not statistically significant. Each additional 3-mo increase in breastfeeding duration was associated with later age at peak velocity (*β* = 0.11 95% CI: 0.01, 0.20) and later age at take-off (*β* = 0.07 95% CI: 0.00, 0.15) adjusting for sex, socioeconomic factors, and maternal and infant characteristics. When expressed relative to the SD of age at peak velocity (±1.09), an additional 9 mo of breastfeeding could delay age at peak velocity by ∼4 mo (∼one-third SD), and an additional year could delay it by ∼6 mo (∼half SD). These differences may be clinically significant, particularly given that delaying puberty onset by just 1 y has been associated with a reduction in adult BMI by 0.38 kg/m^2^ (95% CI: 0.25, 0.51) in females [39], and a 25% lower risk of incident type 2 diabetes in males (Incidence Rate Ratio (IRR) = 0.75 95% CI: 0.57, 0.99 per year) [7].

Previous studies, including 1 in the ECHO-Wide Cohort [22], suggest breastfeeding protects against childhood obesity [23,24,40]. Consistent with this, our results showed that exclusive breastfeeding during the first 3 mo and longer breastfeeding duration were associated with lower BMI *z*-scores at ages 5 and 10 y (Supplementary Table 10), although the effects were modest, particularly at age 5 y. The associations were stronger and statistically significant by age 10 y, suggesting that the inverse association between breastfeeding and childhood adiposity may become more apparent later in childhood. Mediation analyses showed no evidence that childhood BMI mediates the association between breastfeeding and puberty onset. This suggests that the association between breastfeeding and later puberty onset may not be fully explained by reductions in childhood BMI and may potentially involve endocrine or immunologic pathways not measured in this study. For example, breastfeeding appears to influence the growth hormone-insulin-like growth factor 1 (IGF-1) axis [41], and formula-fed infants typically show higher concentrations of IGF-1, a hormone linked to faster growth and earlier pubertal onset [41,42]. Breastfeeding may lead to lower early-life IGF-1 exposure, potentially delaying skeletal growth acceleration and puberty onset. Additionally, the observed association between increased breastfeeding and later onset of puberty could be confounded by maternal factors inversely associated with breastfeeding, such as maternal depression [43]. Data on maternal depression were available, and adjusting for this variable did not alter our findings (Supplementary Table 13).

Our findings align with prior studies examining the relationship between breastfeeding and pubertal onset, often using indicators such as age at menarche in females or age at voice breaking in males [[16], [17], [18],^20^,[44], [45], [46]]. For example, a Danish cohort study reported that boys never exclusively breastfed reached pubertal markers ∼4 mo earlier than those exclusively breastfed ≥4 mo; independent of childhood BMI, though no associations were observed in girls and formal tests for sex differences or mediation by BMI were not conducted [16]. Similarly, a United States study found that girls never breastfed experienced earlier breast and pubic hair development compared with those breastfed ≥6 mo. Although adjustment for prepubertal BMI slightly attenuated the associations, formal mediation analyses were not conducted [18]. Fewer studies have focused on the timing of the adolescent growth spurt in relation to breastfeeding. A Japanese study using the Super-Imposition by Translation and Rotation growth model found that exclusive breastfeeding was associated with later age at peak velocity in girls, and that longer breastfeeding duration was linked to later age at peak velocity in a dose-response manner [19]. Our study adds to the literature by assessing multiple growth parameters. Unlike prior studies limited to age at peak velocity, we used the Preece–Baines growth curve model, which provides a broader range of biologically meaningful parameters [27].

This study has limitations. First, the extant data obtained from the various cohorts collected breastfeeding information using different methods. Thus, the definition of exclusive breastfeeding was not completely consistent and did not capture information on pumped milk. This distinction may be important, as the effects of exclusive breastfeeding can vary depending on whether breast milk is delivered directly from the breast of via bottle. For example, evidence suggests that infants exclusively fed at the breast have a lower risk of becoming overweight compared with those fed expressed breast milk or formula from a bottle [47,48]. Without data differentiating between breastfeeding directly from the breast and bottle-feeding of expressed milk, we are unable to assess the potentially meaningful differences in our analysis. In addition, our exposure variable on exclusive breastfeeding allowed missing or unknown data on supplements and foods, which could potentially increase exposure misclassification. Second, we lacked data on potentially important confounders, such as maternal pubertal timing, which could have influenced our observed associations. Third, BMI is an imperfect measure of adiposity in children. Some studies suggest that increases in BMI at ages 4 to 6.5 y may be primarily driven by changes in lean mass rather than fat mass [49], which may limit the accuracy of our mediation analysis in capturing the role of true adiposity in the association between breastfeeding and pubertal onset. Fourth, the Preece–Baines model tends to underestimate age at take-off slightly, however it provides robust estimates for other key growth parameters [50]. Fifth, nearly 40% of the data on breastfeeding duration was imputed. Nevertheless, complete-case analyses examining the associations between breastfeeding duration and growth parameters yielded similar, and in some cases slightly stronger results than analyses using imputed data. Sixth, participants were largely Black or White, with limited numbers of other ethnicities and a higher proportion of Blacks than the national average, which may limit the generalizability of our findings. Finally, our estimates primarily reflect associations within the shorter range of breastfeeding duration (∼15 wk on average), and the applicability of our findings to populations where longer breastfeeding durations are common, may be limited. This study also has strengths. We leveraged longitudinal data from a United States sample by combining data from multiple ECHO pediatric cohorts. Importantly, we were able to estimate parameters of puberty onset in both males and females. Additionally, the richness of the ECHO data repository allowed for adjustment of key confounding factors, including maternal pregestational BMI and gestational weight gain, thereby strengthening the robustness of our analyses.

Current guidelines recommend exclusive breastfeeding for the first 6 mo of life and continued breastfeeding for ≥2 y, with complementary foods being introduced at age 6 mo [51]. Our results suggest adopting these recommendations may help prevent earlier onset of puberty.

In conclusion, our findings show that breastfeeding, whether exclusive or total duration, was associated with later pubertal onset, suggesting protective effects. Specifically, breastfeeding appears to influence the timing of the adolescent growth spurt by conferring later age at peak velocity and maturation, with rather weak effects on velocity of growth or height ultimately attained. We found no evidence that childhood BMI mediated the associations.

## Author contributions

The authors’ responsibilities were as follows – MJR-L: designed the study, conducted the analyses, interpreted the data, and drafted the initial manuscript; RLH: designed the study, interpreted the data, and critically reviewed the manuscript for important intellectual content; MS: interpreted the data and reviewed the manuscript for important intellectual content; and all authors: approved the final manuscript as submitted and agree to be accountable for all aspects of the work.

## Data availability

Data described in the manuscript, code book, and analytic code will be made available on request pending application and approval at https://dash.nichd.nih.gov/.

## Funding

This research was supported by the Intramural Research Program of the National Institute of Diabetes and Digestive and Kidney Diseases within the NIH. The contributions of the NIH author(s) were made as part of their official duties as NIH federal employees, are in compliance with agency policy requirements, and are considered Works of the United States Government. However, the findings and conclusions presented in this paper are those of the author(s) and do not necessarily reflect the views of the NIH or the United States Department of Health and Human Services.

## Conflict of interest

The authors report no conflicts of interest.
