# Supplementary material for: Associations between breastfeeding, childhood BMI and pubertal onset: findings from a prospective cohort study
Source: Am J Clin Nutr. 2026 Jan 27;123(3):101208. doi: 10.1016/j.ajcnut.2026.101208 (PMC12975361; doi:10.1016/j.ajcnut.2026.101208)
Supplement: Multimedia component 1 [file mmc1.docx]

Associations between breastfeeding, childhood BMI and pubertal onset: Findings from a prospective cohort study

Ramirez-Luzuriaga MJ et al.

[**Supplementary Table 1.** Number of height examinations used to derive Preece-Baines growth parameters 2](#_Toc218077460)

[**Supplementary Table 2**. Demographic characteristics [n (%)] of participants by study cohort 3](#_Toc218077461)

[**Supplementary Table 3.** Characteristics of participants not included in the analyses 4](#_Toc218077462)

[**Supplementary Table 4.** Cross-validation correlations 5](#_Toc218077463)

[**Supplementary Table 5.** Proportion of missing data addressed with multiple imputation 6](#_Toc218077464)

[**Supplementary Table 6**. Logistic missingness models for variables with substantial missingness 7](#_Toc218077465)

[**Supplementary Table 7.** Selected study characteristics [mean ± SD or %] by sex ^1^ 9](#_Toc218077466)

[**Supplementary Table 8.** Complete case analyses of associations of exclusive breastfeeding through 3 months of age (versus no exclusive breastfeeding) with adolescent growth parameters ^1^ 10](#_Toc218077467)

[**Supplementary Table 9.** Complete case analyses of associations of any breastfeeding duration (per 3-month increase) with adolescent growth parameters ^1^ 11](#_Toc218077468)

[**Supplementary Table 10.** Associations of exclusive breastfeeding and breastfeeding duration with childhood BMI ^1^ 12](#_Toc218077469)

[**Supplementary Table 11.** Associations of exclusive breastfeeding through 3 months of age (versus no exclusive breastfeeding) with adolescent growth parameters, stratified by number of exams used to derive the Preece-Baines growth curves ^1^ 13](#_Toc218077470)

[**Supplementary Table 12.** Associations of any breastfeeding duration (per 3-month increase) with adolescent growth parameters, stratified by number of exams used to derive the Preece-Baines growth curves ^1^ 14](#_Toc218077471)

[**Supplementary Table 13.** Associations of exclusive breastfeeding through 3 months of age (versus no exclusive breastfeeding) with adolescent growth parameters accounting for maternal depression ^1^ 15](#_Toc218077472)

[**Supplementary Figure 1** Participant flowchart 16](#_Toc218077473)

[**Supplementary Methods:** Preece-Baines Modeling in the ECHO cohort dataset and missing data patterns 17](#_Toc218077474)

[**References** 20](#_Toc218077475)

# **Supplementary Table 1.** Number of height examinations used to derive Preece-Baines growth parameters

| **Number of repeated measures** | **n** | **%** |
| --- | --- | --- |
| 3 | 18 | 2.94 |
| 4 | 182 | 29.7 |
| 5 | 42 | 6.85 |
| 6 | 267 | 43.6 |
| 7 | 54 | 8.81 |
| 8 | 6 | 0.98 |
| 9 | 3 | 0.49 |
| 10 | 11 | 1.79 |
| 11 | 13 | 2.12 |
| 12 | 8 | 1.31 |
| 13 | 4 | 0.65 |
| 14 | 5 | 0.82 |
| **Total** | **613** | **100** |

# **Supplementary Table 2**. Demographic characteristics [n (%)] of participants by study cohort

|  | **Cohort 1** | **Cohort 2** | **Cohort 3** | **Cohort 4** | **Cohort 5** | **Cohort 6** |
| --- | --- | --- | --- | --- | --- | --- |
|  | **n=186** | **n=371** | **n=50** | **n=2** | **n=2** | **n=2** |
| **Ethnicity** |  |  |  |  |  |  |
| Not Hispanic or Latino | 168 (90.3) | 365 (98.4) | 50 (100) | 2 (100) | 0 (0.00) | 1 (50) |
| Hispanic or Latino | 18 (9.68) | 6 (1.6) | 0 (0.00) | 0 (0.00) | 2 (100) | 1 (50) |
| **Race** |  |  |  |  |  |  |
| White | 131 (70.4) | 176 (47.4) | 0 (0.00) | 2 (100) | 0 (0.00) | 0 (0.00) |
| Black | 32 (17.2) | 192 (51.8) | 49 (98.0) | 0 (0.00) | 0 (0.00) | 0 (0.00) |
| Other | 23 (12.4) | 3 (0.81) | 1 (2.00) | 0 (0.00) | 2 (100) | 2 (100) |
| **Sex** |  |  |  |  |  |  |
| Male | 86 (46.2) | 189 (50.9) | 23 (46.0) | 1 (50) | 2 (100) | 0 (0.00) |
| Female | 100 (53.8) | 182 (49.1) | 27 (54.0) | 1 (50) | 0 (0.00) | 2 (100) |
| **Participant year of birth** |  |  |  |  |  |  |
| 1998-2000 | 106 (56.9) | 0 (0.00) | 0 (0.00) | 0 (0.00) | 0 (0.00) | 0 (0.00) |
| 2001-2003 | 80 (43.0) | 23 (6.20) | 0 (0.00) | 0 (0.00) | 2 (100) | 0 (0.00) |
| 2004-2006 | 0 (0.00) | 348 (93.8) | 50 (100) | 2 (100) | 0 (0.00) | 2 (100) |
| **Participant year of enrollment** |  |  |  |  |  |  |
| 1999-2001 | 173 (93.0) | 0 (0.00) | 0 (0.00) | 0 (0.00) | 0 (0.00) | 0 (0.00) |
| 2002-2003 | 13 (6.99) | 210 (56.6) | 0 (0.00) | 0 (0.00) | 2 (100) | 0 (0.00) |
| 2004-2006 | 0 (0.00) | 161 (43.4) | 50 (100) | 2 (100) | 0 (0.00) | 2 (100) |

# **Supplementary Table 3.** Characteristics of participants not included in the analyses

|  | **Cohort 1** | | **Cohort 2** | | **Cohort 3** | |
| --- | --- | --- | --- | --- | --- | --- |
|  | Did not meet criteria to participate ^1^  (n=223) | Met criteria to participate  (n=186) | Did not meet criteria to participate ^1^  (n=74) | Met criteria to participate  (n=371) | Did not meet criteria to participate ^1^  (n=278) | Met criteria to participate  (n=50) |
| Maternal pre-pregnancy BMI, kg/m^2^ | 24.1 ± 4.77 | 24.9 ± 5.43 | 29.8 ± 7.82 | 27.6 ± 7.58 | 29.1 ± 7.57 | 27.1 ± 7.25 |
| Birthweight, g | 3447 ± 565 | 3502 ± 545 | 3314 ± 612 | 3318 ± 567 | 3220 ± 469 | 3291 ± 565 |
| Birth length, cm | 49.8 ± 2.23 | 49.7 ± 2.37 | 51.1 ± 3.29 | 50.7 ± 3.36 | 50.1 ± 2.45 | 50.5 ± 2.59 |
| **Ethnicity** |  |  |  |  |  |  |
| Not Hispanic or Latino | 207 (92.8) | 168 (90.3) | 72 (97.3) | 366 (98.6) | 226 (81.3) | 50 (100) |
| Hispanic or Latino | 16 (11.2) | 18 (9.68) | 2 (2.70) | 5 (1.35) | 52 (18.7) | 0 (0.00) |
| **Race** |  |  |  |  |  |  |
| White | 167 (74.9) | 131 (70.4) | 40 (54.0) | 176 (47.4) | 2 (0.72) | 0 (0.00) |
| Black | 31 (13.9) | 32 (17.2) | 34 (45.9) | 192 (51.7) | 200 (71.9) | 49 (98.0) |
| Other | 25 (11.2) | 23 (12.4) | 0 (0.00) | 3 (0.81) | 76 (27.3) | 1 (2.0) |
| **Sex** |  |  |  |  |  |  |
| Male | 94 (42.1) | 86 (46.2) | 35 (47.3) | 189 (50.9) | 143 (51.4) | 23 (46.0) |
| Female | 129 (57.8) | 100 (53.8) | 39 (52.7) | 182 (49.1) | 135 (48.6) | 27 (54.0) |

^1^ Criteria consisted of participants within each cohort between 1 to 25 years of age, with available data on infant feeding practices (not restricted to exclusive breastfeeding or breastfeeding duration) and with available height measures (not restricted to at least 3 measures).

# **Supplementary Table 4.** Cross-validation correlations

|  | **Mean** | **Mean difference^1^** | **SD of difference** | **Coefficient of Variation** | **Partial Correlation^2^** |
| --- | --- | --- | --- | --- | --- |
| Age at take-off, yr | 8.42 | 0.03 | 0.37 | 4.43 | 0.87 |
| Age at peak velocity, yr | 11.8 | 0.05 | 0.42 | 3.54 | 0.91 |
| Age at maturation, yr | 16.0 | -0.00 | 0.38 | 2.37 | 0.92 |
| Velocity at take-off, cm/yr | 5.47 | 0.01 | 0.29 | 5.25 | 0.90 |
| Velocity at peak velocity, cm/yr | 7.27 | -0.02 | 0.65 | 8.98 | 0.74 |
| Attained height, cm | 171.1 | -0.02 | 0.87 | 0.51 | 0.99 |
| Height at take-off, cm | 131.3 | 0.19 | 3.18 | 2.43 | 0.88 |
| Height peak velocity, cm | 152.1 | 0.25 | 1.79 | 1.18 | 0.96 |

^1^ Mean difference is the difference estimate derived from 3 examinations minus the estimate from ≥ 5 examinations. ^2^ Correlations between the estimates, adjusted for sex.

# **Supplementary Table 5.** Proportion of missing data addressed with multiple imputation

|  | **n missing** | **% missing** |
| --- | --- | --- |
| Breastfeeding duration | 239 | 38.9 |
| Exclusive breastfeeding through 3 months of age | 81 | 13.2 |
| Maternal age at delivery | 436 | 71.1 |
| Pre-gestational BMI | 262 | 42.7 |
| Maternal height | 170 | 27.7 |
| Mother's highest educational level completed | 387 | 63.1 |
| Annual household income during pregnancy | 406 | 66.2 |
| Gestational age at delivery | 371 | 60.5 |
| Birth length | 84 | 13.7 |
| BMI z-score at age 5 y | 8 | 1.30 |
| BMI z-score at age 10 y | 64 | 10.4 |
| Weight change during pregnancy | 459 | 74.8 |

Abbreviations: BMI, Body Mass Index.

# **Supplementary Table 6**. Logistic missingness models for variables with substantial missingness

|  | **Maximum Likelihood Estimate** | **SE** | **P-value** | **OR** | **95 % CI** |
| --- | --- | --- | --- | --- | --- |
| **Breastfeeding duration (R=1)** |  |  |  |  |  |
| Child’s attained adult height | 0.0069 | 0.0129 | 0.5904 | 1.007 | 0.98, 1.03 |
| Child’s birthweight | 0.1661 | 0.1638 | 0.3105 | 1.181 | 0.85, 1.62 |
| Child’s sex | 0.0888 | 0.2519 | 0.7244 | 1.093 | 0.66, 1.79 |
| Childs data of birth | -0.0009 | 0.0002 | <0.0001 | 0.999 | 0.99, 0.99 |
| Date of visit | 0.0000 | 0.0001 | 0.8869 | 1.000 | 1.00, 1.00 |
| Mom’s race (white) | 0.2075 | 0.4468 | 0.6423 | 1.231 | 0.51, 2.95 |
| Mom’s race (black) | -0.5683 | 0.4574 | 0.2141 | 0.566 | 0.23, 1.38 |
| **Maternal age at delivery (R=1)** |  |  |  |  |  |
| Child’s attained adult height | -0.0324 | 0.0339 | 0.3387 | 0.968 | 0.91, 1.03 |
| Child’s birthweight | 0.1896 | 0.4331 | 0.6615 | 1.209 | 0.52, 2.82 |
| Child’s sex | -1.4441 | 0.6358 | 0.0231 | 0.236 | 0.07, 0.82 |
| Childs data of birth | -0.0073 | 0.0007 | <0.0001 | 0.993 | 0.99, 0.99 |
| Date of visit | 0.0002 | 0.0003 | 0.6394 | 1.000 | 1.00, 1.00 |
| Mom’s race (white) | -0.6434 | 0.8244 | 0.4351 | 0.526 | 0.10, 2.64 |
| Mom’s race (black) | -1.5544 | 0.8839 | 0.0786 | 0.211 | 0.03, 1.19 |
| **Pre-gestational BMI (R=1)** |  |  |  |  |  |
| Child’s attained adult height | -0.0113 | 0.0128 | 0.3753 | 0.989 | 0.96, 1.01 |
| Child’s birthweight | 0.0620 | 0.1626 | 0.7031 | 1.064 | 0.77,1.46 |
| Child’s sex | -0.1449 | 0.2495 | 0.5615 | 0.865 | 0.53, 1.41 |
| Childs data of birth | -0.0009 | 0.0002 | <0.0001 | 0.999 | 0.99, 0.99 |
| Date of visit | -0.0003 | 0.0001 | 0.0087 | 1.000 | 1.00, 1.00 |
| Mom’s race (white) | -0.9249 | 0.5649 | 0.1015 | 0.397 | 0.13, 1.20 |
| Mom’s race (black) | -1.3015 | 0.5734 | 0.0232 | 0.272 | 0.08, 0.83 |
| **Mothers highest educational level completed (R=1)** |  |  |  |  |  |
| Child’s attained adult height | -0.0373 | 0.0154 | 0.0156 | 0.963 | 0.93, 0.99 |
| Child’s birthweight | 0.3388 | 0.1926 | 0.0785 | 1.403 | 0.96, 2.04 |
| Child’s sex | -0.6472 | 0.2966 | 0.0291 | 0.523 | 0.29, 0.93 |
| Childs data of birth | -0.0021 | 0.0002 | <0.0001 | 0.998 | 0.99, 0.99 |
| Date of visit | -0.0005 | 0.0001 | <0.0001 | 1.000 | 0.99, 1.00 |
| Mom’s race (white) | -1.4694 | 0.5463 | 0.0072 | 0.230 | 0.07, 0.67 |
| Mom’s race (black) | -0.9045 | 0.5596 | 0.1060 | 0.405 | 0.13, 1.21 |
| **Annual household income during pregnancy (R=1)** |  |  |  |  |  |
| Child’s attained adult height | -0.0307 | 0.0148 | 0.0385 | 0.970 | 0.94, 0.99 |
| Child’s birthweight | 0.3368 | 0.1826 | 0.0651 | 1.400 | 0.97, 2.00 |
| Child’s sex | -0.4808 | 0.2819 | 0.0881 | 0.618 | 0.35, 1.07 |
| Childs data of birth | -0.0013 | 0.0002 | <0.0001 | 0.999 | 0.99, 0.99 |
| Date of visit | -0.0005 | 0.0001 | <0.0001 | 1.000 | 0.99, 1.00 |
| Mom’s race (white) | -0.5434 | 0.4240 | 0.2000 | 0.581 | 0.25, 1.33 |
| Mom’s race (black) | -0.3888 | 0.4464 | 0.3838 | 0.678 | 0.28, 1.62 |
| **Gestational age at delivery (R=1)** |  |  |  |  |  |
| Child’s attained adult height | -0.0278 | 0.0158 | 0.0788 | 0.973 | 0.94, 1.00 |
| Child’s birthweight | 0.3619 | 0.2004 | 0.0710 | 1.436 | 0.97, 2.12 |
| Child’s sex | -0.2808 | 0.3052 | 0.3576 | 0.755 | 0.41, 1.37 |
| Childs data of birth | -0.0024 | 0.0003 | <0.0001 | 0.998 | 0.99, 0.99 |
| Date of visit | -0.0006 | 0.0001 | <0.0001 | 0.999 | 0.99, 1.00 |
| Mom’s race (white) | -2.3593 | 0.7109 | 0.0009 | 0.094 | 0.02, 0.38 |
| Mom’s race (black) | -1.6991 | 0.7146 | 0.0174 | 0.183 | 0.04, 0.74 |
| **Weight change during pregnancy (R=1)** |  |  |  |  |  |
| Child’s attained adult height | -0.0039 | 0.0156 | 0.8051 | 0.996 | 0.96, 1.02 |
| Child’s birthweight | 0.0306 | 0.2069 | 0.8823 | 1.031 | 0.68, 1.54 |
| Child’s sex | -0.2113 | 0.3187 | 0.5074 | 0.810 | 0.43, 1.51 |
| Childs data of birth | 0.0048 | 0.0005 | <0.0001 | 1.005 | 1.00, 1.00 |
| Date of visit | -0.0001 | 0.0001 | 0.2640 | 1.000 | 1.00, 1.00 |
| Mom’s race (white) | -0.4448 | 0.7640 | 0.5605 | 0.641 | 0.14, 2.86 |
| Mom’s race (black) | -1.1867 | 0.7776 | 0.1270 | 0.305 | 0.06, 1.40 |

The probability of an observed value (R=1) for each variable was modeled as a function of observed maternal characteristics, child characteristics, sociodemographic indicators and procedural variables related to data collection. Sampe size in all models was 613.

# **Supplementary Table 7.** Selected study characteristics [mean ± SD or %] by sex ^1^

|  | **n** | **Females** | **n** | **Males** |
| --- | --- | --- | --- | --- |
| Breastfeeding characteristics |  |  |  |  |
| Exclusive breastfeeding through 3 months of age, % ^2^ |  |  |  |  |
| Yes | 30 | 9.62 | 53 | 17.6 |
| No | 282 | 90.4 | 248 | 82.4 |
| Duration of any breastfeeding, wks. |  |  |  |  |
| Mean ± SD | 312 | 14.8 ± 14.3 | 301 | 15.7 ± 14.6 |
| Median (25^th^, 75^th^ percentile) | 312 | 11.5 (3.19, 22.3) | 301 | 12.6 (3.81, 22.5) |
| Range, wks. | 312 | 0-78 | 301 | 0-69 |
| Breastfeeding duration, % |  |  |  |  |
| < 12 weeks | 158 | 50.5 | 141 | 47.0 |
| 12 to 24 weeks | 69 | 22.1 | 75 | 25.0 |
| > 24 weeks | 85 | 27.4 | 85 | 28.0 |
| Offspring adolescent growth parameters |  |  |  |  |
| Age at take-off, y | 312 | 7.70 ± 0.82 | 301 | 9.03 ± 0.67 |
| Age at peak velocity, y | 312 | 10.5 ± 1.09 | 301 | 12.8 ± 0.91 |
| Age at maturation, y | 312 | 14.9 ± 0.90 | 301 | 17.0 ± 0.98 |
| Velocity at take-off, cm/y | 312 | 5.83 ± 0.76 | 301 | 5.23 ± 0.49 |
| Peak velocity, cm/y | 312 | 6.74 ± 0.73 | 301 | 7.72 ± 1.04 |
| Attained height, cm | 312 | 163.7 ± 6.70 | 301 | 177.9 ± 7.47 |
| Height at take-off, cm | 312 | 127.5 ± 6.77 | 301 | 134.7 ± 6.57 |
| Height at peak velocity, cm | 312 | 144.8 ± 5.77 | 301 | 158.8 ± 6.73 |

^1^ Values are mean ± SD or % unless specified otherwise.

# **Supplementary Table 8.** Complete case analyses of associations of exclusive breastfeeding through 3 months of age (versus no exclusive breastfeeding) with adolescent growth parameters ^1^

|  | **Pooled (n=528)** | | | **Females (n=270)** | | | **Males (n=258)** | | |
| --- | --- | --- | --- | --- | --- | --- | --- | --- | --- |
|  | **Model 1** | **Model 2** | **Model 3** | **Model 1** | **Model 2** | **Model 3** | **Model 1** | **Model 2** | **Model 3** |
| Age at take-off, yr | 0.22 (-0.02, 0.46) | 0.19 (-0.04, 0.44) | 0.18 (-0.05, 0.43) | 0.22 (-0.18, 0.62) | 0.17 (-0.12, 0.46) | 0.22 (-0.18, 0.62) | 0.23 (-0.04, 0.51) | 0.17 (-0.13, 0.47) | 0.15 (-0.13, 0.45) |
| Age at peak velocity, yr | 0.38 (0.06, 0.70) | 0.37 (0.04, 0.69) | 0.33 (0.01, 0.64) | 0.32 (-0.19, 0.85) | 0.32 (-0.19, 0.85) | 0.28 (-0.22, 0.79) | 0.48 (0.10, 0.86) | 0.41 (0.01, 0.81) | 0.37 (-0.00, 0.76) |
| Age at maturation, yr | 0.36 (0.06, 0.66) | 0.31 (0.00, 0.62) | 0.28 (-0.01, 0.58) | 0.27 (-0.16, 0.71) | 0.28 (-0.16, 0.72) | 0.24 (-0.18, 0.68) | 0.46 (0.04, 0.88) | 0.34 (-0.09, 0.77) | 0.31 (-0.11, 0.74) |
| Velocity at take-off, cm/yr | -0.12 (-0.32, 0.07) | -0.11 (-0.32, 0.08) | -0.10 (-0.30, 0.10) | -0.16 (-0.52, 0.20) | -0.17 (-0.54, 0.17) | -0.15 (-0.51, 0.20) | -0.06 (-0.27,0.13) | -0.06 (-0.27, 0.14) | -0.05 (-0.25, 0.15) |
| Peak velocity, cm/yr | 0.11 (-0.17, 0.41) | 0.11 (-0.17, 0.41) | 0.10 (-0.18, 0.40) | -0.11 (-0.48, 0.24) | -0.14 (-0.50, 0.21) | -0.14 (-0.50, 0.21) | 0.28 (-0.16, 0.74) | 0.33 (-0.14, 0.81) | 0.30 (-0.17, 0.77) |
| Attained height, cm | 1.64 (-0.60, 3.89) | 0.84 (-1.44, 3.13) | 0.88 (-1.41, 3.17) | 0.52 (-2.73, 3.78) | -0.03 (-3.22, 3.16) | 0.00 (-3.20, 3.20) | 2.67 (-0.48, 5.82) | 1.73 (-1.36, 4.84) | 1.77 (-1.33, 4.88) |
| Height at take-off, cm | 0.57 (-1.52, 2.67) | -0.01 (2.15, 2.12) | 0.15 (-1.96, 2.27) | 0.77 (-2.51, 4.06) | 0.51 (-2.73, 3.76) | 0.67 (-2.52, 3.87) | 0.45 (-2.26, 3.18) | -0.17 (-2.90, 2.54) | 0.02 (-2.67, 2.71) |
| Height at peak velocity, cm | 1.64 (-0.34, 3.63) | 0.99 (-1.00, 3.00) | 1.01 (-0.99, 3.02) | 1.08 (-1.74, 3.91) | 0.64 (-2.10, 3.39) | 0.62 (-2.13, 3.38) | 2.28 (-0.54, 5.11) | 1.52 (-1.27, 4.32) | 1.57 (-1.22, 4.37) |

^1^ Estimates are β coefficients (95% CI) from linear regression models. **Model 1** accounts for child’s date of birth, race, and cohort id. **Model 2** additionally accounts for maternal education, annual household income during pregnancy, maternal age at delivery, maternal height, maternal pre-pregnancy BMI, total gestational weight change, gestational age at delivery, birthweight, and birth length. **Model 3.** Additionally accounts for child BMI z-score at age 5. Pooled models additionally adjust for sex. Multiple imputation techniques were used for missing covariates.

# **Supplementary Table 9.** Complete case analyses of associations of any breastfeeding duration (per 3-month increase) with adolescent growth parameters ^1^

|  | **Pooled (n=374)** | | | **Females (n=191)** | | | **Males (n=183)** | | |
| --- | --- | --- | --- | --- | --- | --- | --- | --- | --- |
|  | **Model 1** | **Model 2** | **Model 3** | **Model 1** | **Model 2** | **Model 3** | **Model 1** | **Model 2** | **Model 3** |
| Age at take-off, yr | 0.07 (0.02, 0.13) | 0.08 (0.01, 0.14) | 0.07 (0.00, 0.13) | 0.13 (0.04, 0.21) | 0.14 (0.03, 0.24) | 0.13 (0.02, 0.23) | 0.03 (-0.03, 0.10) | 0.01 (-0.07, 0.09) | 0.00 (-0.07, 0.09) |
| Age at peak velocity, yr | 0.11 (0.04, 0.19) | 0.12 (0.03, 0.21) | 0.09 (0.00, 0.18) | 0.17 (0.06, 0.28) | 0.19 (0.05, 0.33) | 0.16 (0.03, 0.30) | 0.05 (-0.04, 0.14) | 0.04 (-0.07, 0.15) | 0.01 (-0.09, 0.13) |
| Age at maturation, yr | 0.09 (0.02, 0.16) | 0.09 (0.01, 0.18) | 0.06 (-0.01, 0.15) | 0.14 (0.04, 0.23) | 0.15 (0.03, 0.27) | 0.13 (0.02, 0.25) | 0.04 (-0.05, 0.14) | 0.02 (-0.09, 0.14) | 0.00 (-0.11, 0.12) |
| Velocity at take-off, cm/yr | -0.04 (-0.08, 0.00) | -0.05 (-0.11, -0.00) | -0.04 (-0.09, 0.01) | -0.10 (-0.18, -0.01) | -0.11 (-0.21,  -0.02) | -0.10 (-0.19, -0.01) | 0.01 (-0.03, 0.06) | 0.00 (-0.05, 0.06) | 0.01 (-0.04, 0.07) |
| Peak velocity, cm/yr | -0.00 (-0.06, 0.06) | -0.00 (-0.08, 0.07) | -0.01 (-0.09, 0.06) | -0.08 (-0.16, -0.00) | -0.09 (-0.19, -0.00) | -0.10 (-0.19, -0.00) | 0.06 (-0.03, 0.17) | 0.07 (-0.05, 0.20) | 0.06 (-0.07, 0.19) |
| Attained height, cm | 0.48 (-0.06, 1.02) | 0.13 (-0.46, 0.73) | 0.17 (-0.43, 0.79) | 0.12 (-0.60, 0.85) | -0.11 (-0.86, 0.64) | -0.06 (-0.83, 0.69) | 0.72 (-0.09, 1.54) | 0.22 (-0.73, 1.18) | 0.27 (-0.69, 1.24) |
| Height at take-off, cm | 0.42 (-0.08, 0.92) | 0.13 (-0.44, 0.70) | 0.25 (-0.32, 0.83) | 0.42 (-0.27, 1.13) | 0.26 (-0.52, 1.05) | 0.35 (-0.43, 1.14) | 0.38 (-0.36, 1.12) | -0.11 (-1.00, 0.78) | 0.04 (-0.83, 0.92) |
| Height at peak velocity, cm | 0.58 (0.10, 1.06) | 0.28 (-0.25, 0.82) | 0.31 (-0.23, 0.85) | 0.44 (-0.17, 1.06) | 0.28 (-0.38, 0.95) | 0.28 (-0.39, 0.96) | 0.62 (-0.12, 1.37) | 0.15 (-0.70, 1.02) | 0.21 (-0.65, 1.09) |

^1^ Estimates are β coefficients (95% CI) from linear regression models. **Model 1** accounts for child’s date of birth, race, and cohort id. **Model 2** additionally accounts for maternal education, annual household income during pregnancy, maternal age at delivery, maternal height, maternal pre-pregnancy BMI, total gestational weight change, gestational age at delivery, birthweight, and birth length. **Model 3** Additionally accounts for child BMI z-score at age 5. Pooled models additionally adjust for sex. Multiple imputation techniques were used for missing covariates.

# **Supplementary Table 10.** Associations of exclusive breastfeeding and breastfeeding duration with childhood BMI ^1^

|  | **Imputed** | | **Complete case analyses** | |
| --- | --- | --- | --- | --- |
|  | **Exclusive breastfeeding through 3 months of age (vs no exclusive BF)**  **(n=613)** | **Breastfeeding duration (per 3-months increase)**  **(n=613)** | **Exclusive breastfeeding through 3 months of age (vs no exclusive BF)**  **(n=528)** | **Breastfeeding duration** **(per 3-months increase)**  **(n=374)** |
| BMI z-score at age 5 |  |  |  |  |
| Model 1 | -0.22 (-0.51, 0.07) | -0.08 (-0.17, 0.00) | -0.28 (-0.63, 0.07) | -0.09 (-0.17, -0.01) |
| Model 2 | -0.21 (-0.52, 0.09) | -0.08 (-0.17, 0.01) | -0.29 (-0.64, 0.06) | -0.09 (-0.18, -0.00) |
| BMI z-score at age 10 |  |  |  |  |
| Model 1 | -0.40 (-0.69, -0.10) | -0.09 (-0.18, -0.00) | -0.49 (-0.83, -0.14) | -0.11 (-0.19, -0.02) |
| Model 2 | -0.26 (-0.57, 0.03) | -0.08 (-0.17, 0.00) | -0.32 (-0.68, 0.03) | -0.10 (-0.20, -0.00) |

Abbreviations: BMI, Body Mass Index; BF, Breastfeeding

^1^ Estimates are β coefficients (95% CI) from linear regression models. **Model 1** adjusts for age at time of measurement and sex. **Model 2** additionally accounts for maternal race, maternal education, socioeconomic status during pregnancy, maternal age at delivery. Multiple imputation techniques were used for missing covariates.

# **Supplementary Table 11.** Associations of exclusive breastfeeding through 3 months of age (versus no exclusive breastfeeding) with adolescent growth parameters, stratified by number of exams used to derive the Preece-Baines growth curves ^1^

|  | **Pooled results**  **(n=613)** | **Pooled results stratified by number of exams (n=613)** | | **P-value for interaction**  **of exclusive breastfeeding with # of exams** |
| --- | --- | --- | --- | --- |
|  |  | **< 5 exams**  **(n=201)** | **≥ 5 exams**  **(n=412)** |  |
| Age at take-off, yr | 0.23 (0.03, 0.42) | 0.25 (0.02, 0.48) | 0.22 (-0.09, 0.53) | 0.673 |
| Age at peak velocity, yr | 0.35 (0.09, 0.61) | 0.38 (0.07, 0.70) | 0.33 (-0.06, 0.74) | 0.830 |
| Age at maturation, yr | 0.34 (0.10, 0.59) | 0.37 (0.08, 0.66) | 0.31 (-0.05, 0.68) | 0.900 |
| Velocity at take-off, cm/yr | -0.08 (-0.25, 0.08) | -0.11 (-0.31, 0.08) | -0.05 (-0.32, 0.20) | 0.669 |
| Peak velocity, cm/yr | 0.07 (-0.15, 0.30) | 0.09 (-0.16, 0.34) | 0.07 (-0.29, 0.43) | 0.168 |
| Attained height, cm | 1.66 (-0.18, 3.52) | 1.66 (-0.68, 4.02) | 1.90 (-0.81,4.63) | 0.741 |
| Height at take-off, cm | 0.91 (-0.82, 2.65) | 0.84 (-1.28, 2.97) | 1.20 (-1.40,3.82) | 0.504 |
| Height at peak velocity, cm | 1.62 (-0.02, 3.26) | 1.61 (-0.42, 3.65) | 1.88 (-0.53, 4.30) | 0.946 |

^1^ Estimates are β coefficients (95% CI) from linear regression models. Models account for child’s date of birth, race, cohort id and sex.

# **Supplementary Table 12.** Associations of any breastfeeding duration (per 3-month increase) with adolescent growth parameters, stratified by number of exams used to derive the Preece-Baines growth curves ^1^

|  | **Pooled results**  **(n=613)** | **Pooled results stratified by number of exams (n=613)** | | **P-value for interaction of breastfeeding duration with # of exams** |
| --- | --- | --- | --- | --- |
|  |  | **< 5 exams**  **(n=201)** | **≥ 5 exams**  **(n=412)** |  |
| Age at take-off, yr | 0.08 (0.02, 0.15) | 0.11 (-0.01, 0.23) | 0.05 (-0.02, 0.11) | 0.362 |
| Age at peak velocity, yr | 0.12 (0.04, 0.19) | 0.12 (-0.05, 0.29) | 0.08 (-0.00, 0.16) | 0.862 |
| Age at maturation, yr | 0.09 (0.02, 0.17) | 0.14 (-0.02, 0.29) | 0.06 (-0.02, 0.14) | 0.533 |
| Velocity at take-off, cm/yr | -0.04 (-0.10, 0.00) | -0.02 (-0.13, 0.08) | -0.03 (-0.08, 0.03) | 0.968 |
| Peak velocity, cm/yr | -0.00 (-0.08, 0.07) | -0.03 (-0.17, 0.11) | 0.01 (-0.07, 0.08) | 0.260 |
| Attained height, cm | 0.44 (-0.12, 1.01) | 0.93 (-0.35, 2.23) | 0.30 (-0.31, 0.91) | 0.424 |
| Height at take-off, cm | 0.44 (-0.09, 0.98) | 0.94 (-0.21, 2.09) | 0.22 (-0.35, 0.80) | 0.131 |
| Height at peak velocity, cm | 0.56 (0.06, 1.06) | 0.92 (-0.18, 2.02) | 0.37 (-0.18, 0.91) | 0.361 |

^1^ Estimates are β coefficients (95% CI) from linear regression models. Models account for child’s date of birth, race, cohort id and sex.

# **Supplementary Table 13.** Associations of exclusive breastfeeding through 3 months of age (versus no exclusive breastfeeding) with adolescent growth parameters accounting for maternal depression ^1^

|  | **Exclusive breastfeeding through 3 months of age (vs no exclusive breastfeeding)** | | | **Breastfeeding duration (per 3-months increase)** | |
| --- | --- | --- | --- | --- | --- |
|  | **n=613** | | | **n=613** | |
|  | **Model 1** | **Model 2** | **Model 1** | | **Model 2** |
| Age at take-off, yr | 0.21 (0.00, 0.42) | 0.19 (-0.01, 0.40) | 0.08 (0.00, 0.16) | | 0.07 (-0.00, 0.15) |
| Age at peak velocity, yr | 0.33 (0.05, 0.61) | 0.29 (0.02, 0.56) | 0.11 (0.02, 0.21) | | 0.09 (0.00, 0.19) |
| Age at maturation, yr | 0.31 (0.05, 0.57) | 0.28 (0.02, 0.53) | 0.09 (0.00, 0.18) | | 0.08 (-0.00, 0.16) |
| Velocity at take-off, cm/yr | -0.09 (-0.27, 0.09) | -0.07 (-0.24, 0.10) | -0.05 (-0.11, 0.01) | | -0.04 (-0.10, 0.02) |
| Peak velocity, cm/yr | 0.08 (-0.16, 0.34) | 0.07 (-0.18, 0.33) | 0.00 (-0.08, 0.09) | | -0.00 (-0.09, 0.09) |
| Attained height, cm | 1.24 (-0.63, 3.11) | 1.27 (-0.60, 3.14) | 0.34 (-0.26, 0.94) | | 0.35 (-0.25, 0.97) |
| Height at take-off | 0.52 (-1.18, 2.24) | 0.69 (-1.01, 2.39) | 0.29 (-0.25, 0.84) | | 0.37 (-0.17, 0.92) |
| Height at peak velocity | 1.21 (-0.41, 2.84) | 1.21 (-0.40, 2.84) | 0.44 (-0.07, 0.96) | | 0.44 (-0.07, 0.97) |

^1^ Estimates are β coefficients (95% CI) from linear regression models. **Model 1** accounts for sex, child’s date of birth, race, cohort id, maternal education, annual household income during pregnancy, maternal age at delivery, maternal height, maternal pre-pregnancy BMI, total gestational weight change, gestational age at delivery, birthweight, and birth length and maternal depression. **Model 2.** Additionally accounts for child BMI z-score at age 5. Multiple imputation techniques were used for missing covariates.

# **Supplementary Figure 1** Participant flowchart

All ECHO children consented

N=30,904 (69 cohorts)

69

All children with data on breastfeeding practices

N= 8,360 children (38 cohorts)

All participants (0-25 years) with anthropometric data

N= (20,126 participants; 76,045 examinations) (54 cohorts)

All participants with ≥ 3 height measures: ≥ 1 measure at ages 1-9 years, ≥ 1 measure at ages 10-15 and ≥ 1 measure at ages 16 years and up

N= 1,100 participants (10 cohorts)

Excluded participants:

Growth parameters failed to be derived from the Preece-Baines model, n=28

Participants with adolescent growth spurt parameters

N= 1,072 participants (10 cohorts)

Participants included in final analyses for associations of breastfeeding duration with adolescent growth spurt parameters.

n=613 children (6 cohorts)

# **Supplementary Methods:** Preece-Baines Modeling in the ECHO cohort dataset and missing data patterns

**Subjects:**

Data were derived from height measures obtained from the ECHO-wide cohort study, part of the Environmental Influences on Child Health Outcomes (ECHO) Program (1). Optimal estimates of the growth parameters are obtained if height measures are made before, during and after the growth spurt. We derived growth parameters in participants with at least 3 height measurements, at least one of which was made at age 1-9, at least one at age 10-15 and at least one at age 16 up. We excluded individuals with an exam that was more than 4 SD away from the age and sex specific height mean to minimize possible outliers. Individuals who had an exam where the height decreased by more than 5 cm from their previous exam were also excluded since these are probably errors. This resulted in 571 females and 501 males, 1072 individuals altogether.

**Preece-Baines model:** The data were fit using the Preece-Baines growth model (model 1 of Preece and Baines)(2) This is a parametric growth model that describes growth from childhood to adulthood in terms of 5 parameters: *h_1_*, the height ultimately attained in adulthood (measured here in cm); *s_0_*, a dimensionless exponential constant representing the growth rate in childhood (*i.e.,* before the adolescent growth spurt); *s_1_*, a dimensionless exponential constant for the growth rate during the growth spurt, *θ*, a time constant that serves to locate age at the middle of the growth spurt (in years), and *h_θ_*, the height at age *θ*. Height at a given age (*t*) is modeled as a compound logistic function:

$h\left( t \right)=h_{1}- \frac{2(h_{1}-h_{\theta})}{e^{[s_{0}\left( t-\theta\right)]}+e^{[s_{1}\left( t-\theta\right)]}}$

Classically, the model has been fit with iterative least-squares procedures separately for each individual to obtain estimates of the parameters for each individual. This requires a large number of examinations for each individual to obtain reliable estimates (usually >8-10). Here we used a non-linear “mixed” model approach, which allows for fitting the model with fewer examinations (3). The mixed model was fit with PROC NLMIXED in SAS (Cary, NC, version 9.4) and the “multiplicative” model was fit which included fixed effects for each of the 5 parameters (representative of the mean values in the population) and subject-specific random effects (representing the variances of each of the parameters under the assumption of multivariate normality) along with their covariances (4, 5). To increase computational speed the “first order” estimation option was used, and to enhance the search for a global maximum likelihood a range of different starting values was used for each parameter- the best-fitting model was chosen over 972 different combinations of starting values.

For the best-fitting model, values of each of the five parameters for each individual were obtained by summing the solutions of the fixed effects and the random effects. With these parameters, developmentally important features of growth can be calculated for each individual either directly from the solutions of the Preece-Baines model (6) or from analysis of the predicted heights across age. These features include: velocity at the initiation of the adolescent growth spurt, *i.e.*, velocity at “take-off” (v_to_), age at take off (age_to_ ) height at take-off (h_to_), peak velocity (pv), age at peak velocity (age_pv_) and height at peak velocity (h_pv_). For the present study, we conducted analyses using both the direct solutions and the analysis of predicted heights and obtained similar results. The analyses presented were obtained using the direct solutions as given in the PBREG Stats module (7), which are mathematically equivalent to those given by Guo et al (8). Age at maturation was taken at the age at which the predicted growth velocity fell below 1 cm/yr (9).

**Cross-validation analysis:** To determine the robustness of the modeling approach to sparse data, e.g., fitting the curve with only 3 examinations, we conducted a cross-validation analysis. Individuals with ≥5 examinations were divided into 10 equally-sized groups and data were masked for all but 3 suitably-spaced examinations and the Preece-Baines model was re-fit for each group. The resulting analyses showed that estimates using only 3 examinations were highly consistent with those using ≥ 5 examinations (Supplementary Table 4). As simulation studies have shown that, even without the mixed model, the model performs well with 5 examinations (where it is fully identifiable) (10), this supports our use of the model as implemented.

**Missing data patterns:**  To evaluate the mechanism underlying missing data, we conducted logistic regression models in which the probability that each variable with substantial missingness was observed was modeled as a function of observed maternal, infant, sociodemographic and procedural characteristics. For each variable, we included child sex, birthweight, attained height, maternal race, and two time-related process variables (child’s date of birth and date of visit) to identify systematic patterns in data availability. Across all models, the most consistent and strongest predictors of missingness were the time-related procedural variables (p < 0.0001), indicating that missingness predominantly reflected administrative or temporal features of data collection. Maternal or child demographic characteristics were generally not significant predictors, and when significant, effects were small and inconsistent across models (see Supplementary Table 6). This pattern supports a Missing at Random (MAR) mechanism driven by observable process-related factors.

# **References**

1 Knapp, E.A., Kress, A.M., Parker, C.B., Page, G.P., McArthur, K., Gachigi, K.K., Alshawabkeh, A.N., Aschner, J.L., Bastain, T.M. and Breton, C.V. (2023) The environmental influences on child health outcomes (ECHO)-wide cohort. *American journal of epidemiology*, **192**, 1249-1263.

2 Preece, M.A. and Baines, M.J. (1978) A new family of mathematical models describing the human growth curve. *Annals of human biology*, **5**, 1-24.

3 Tu, Y.K. and Pandis, N. (2013) Analyzing longitudinal orthodontic data. Part 3: multilevel nonlinear growth models. *American journal of orthodontics and dentofacial orthopedics : official publication of the American Association of Orthodontists, its constituent societies, and the American Board of Orthodontics*, **144**, 779-782.

4 Susman, E.P., Murphy, J.R., Zerbe, G.O. and Jones, R.H. (1998) Using a nonlinear mixed model to evaluate three models of human stature. *Growth, development, and aging : GDA*, **62**, 161-171.

5 Grimm, K.J., Ram, N. and Hamagami, F. (2011) Nonlinear growth curves in developmental research. *Child development*, **82**, 1357-1371.

6 Sayers, A., Baines, M. and Tilling, K. (2013) A new family of mathematical models describing the human growth curve-Erratum: direct calculation of peak height velocity, age at take-off and associated quantities. *Annals of human biology*, **40**, 298-299.

7 Sayers, A. (2013) PBREG: Stata module to fit the Preece and Baines (1978) family of growth curves and age, height, and velocity at peak height velocity. in press.

8 Guo, S., Siervogel, R.M., Roche, A.F. and Chumlea, W.C. (1992) Mathematical modelling of human growth: A comparative study. *American Journal of Human Biology*, **4**, 93-104.

9 Di Giovanni, I., Marcovecchio, M.L., Chiavaroli, V., de Giorgis, T., Chiarelli, F. and Mohn, A. (2017) Being born large for gestational age is associated with earlier pubertal take-off and longer growth duration: a longitudinal study. *Acta paediatrica (Oslo, Norway : 1992)*, **106**, 61-66.

10 Simpkin, A. J., Howe, L. D., Tilling, K., Gaunt, T. R., Lyttleton, O., McArdle, W. L., ... & Relton, C. L. (2017). The epigenetic clock and physical development during childhood and adolescence: longitudinal analysis from a UK birth cohort. *International journal of epidemiology*, *46*(2), 549-558.
